# Involuntay hospitalization for purpose of treatment - experiences and viewpoints from patients with schizophrenia

**DOI:** 10.1192/j.eurpsy.2025.1507

**Published:** 2025-08-26

**Authors:** S. K. Linstow, A. Urfer-Parnas

**Affiliations:** 1 Mental Health Center Amager, København, Denmark

## Abstract

**Introduction:**

Coercive methods in psychiatry have been a matter of intense public debate for many years. Involuntary hospitalization (IH) for purpose of treatment is a major intervention with the purpose of providing care for individuals, who, during psychotic episodes, are not immanently dangerous to self or others but unable to take care of themselves and at risk of significant deterioration. The intervention is, however, not yet fully examined from patients’ perspectives.

**Objectives:**

To examine views and experiences of patients with schizophrenia, involuntary hospitalized in a psychotic state for purpose of treatment.

**Methods:**

Nine patients were interviewed at discharge with a semi-structured instrument on the following: If IH can be justified in general and in the context of their own admission, how IH can be prevented, and finally, how they would react if confronted with a person in a similar condition as their own as described in their chart at the time of IH. The patients were reinterviewed after the interviewer had read their chart to obtain their reactions on others’ descriptions of their condition.

**Results:**

None of the patients considered their involuntary hospitalization necessary in its entirety or as an act of caregiving, and they believed that community support could have prevented it. Some described improvement in their condition attributed not to the hospitalization itself but to positive interactions with staff and other patients. They did not view their condition as psychotic but rather as angry, stressed, or even entirely well. They stressed that psychiatric patients should be able to refuse treatment in the same way as patients with somatic illnesses can.

**Conclusions:**

We discussed the patients’ experiences and negative view of IH, how their opinions can be related to the concept of psychosis and insight, possibilities of increased community support, and ethical issues concerning caregiving when the person being cared for does not feel a need. A better understanding of the role of psychopathology and patients’ subjective experiences may provide a foundation for a patient-doctor dialogue on joint interventions in the future. More options for community support and acute outpatient interventions could be a possible way to reduce IH of patients, who are not dangerous to self or others.

**Disclosure of Interest:**

None Declared

